# Supplementary material for: Patient-derived pancreas-on-a-chip to model cystic fibrosis-related disorders
Source: Nat Commun. 2019 Jul 16;10:3124. doi: 10.1038/s41467-019-11178-w (PMC6635497; doi:10.1038/s41467-019-11178-w)
Supplement: Supplementary file 1 — Supplementary Information [file 41467_2019_11178_MOESM1_ESM.pdf]

Supplementary Information

“Patient-derived pancreas-on-a-chip to model cystic fibrosis-related disorders”

Mun et al.

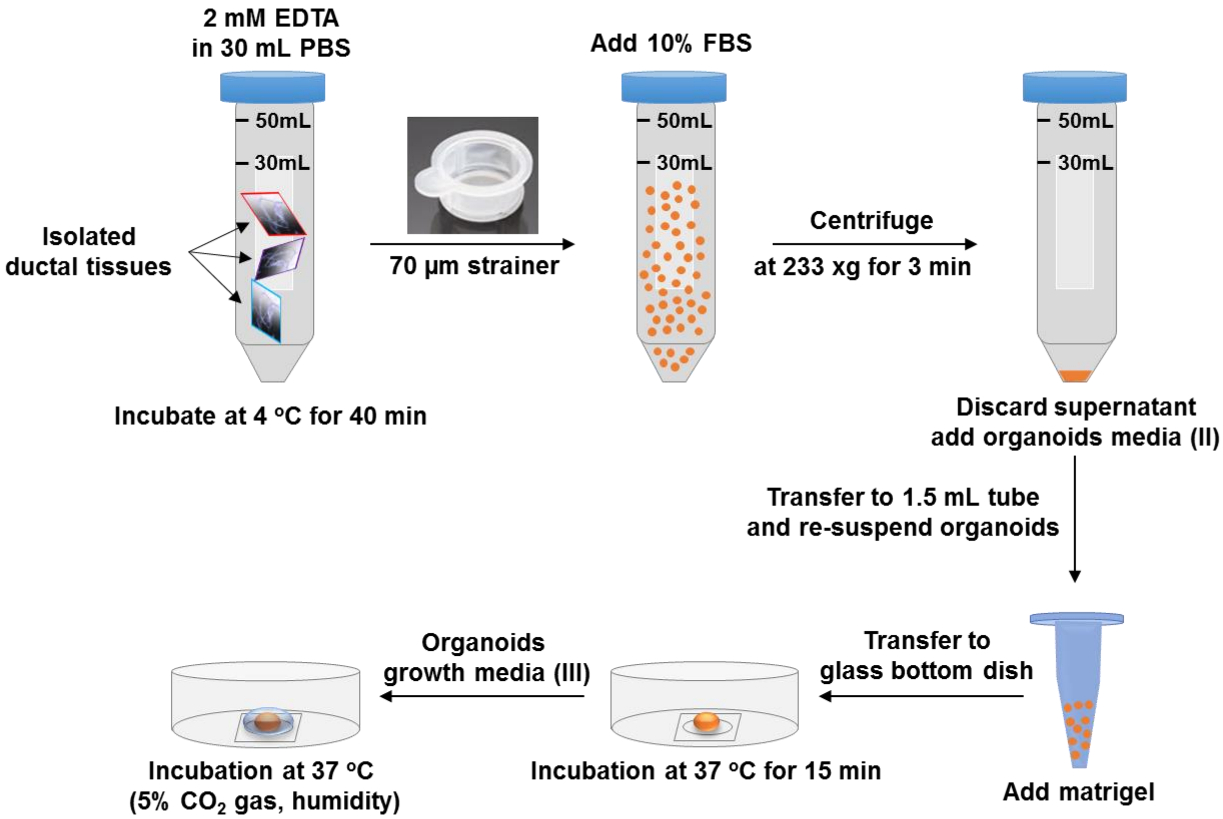

Supplementary Figure 1. Isolation of pancreatic ductal organoids. A schematic shows the isolation process of pancreatic ductal organoids from pancreatic ductal tissue. The pancreatic duct is digested to take PDECs out from the tissue and PDECs are spun down after filtering. PDECs are embedded in Matrigel and covered by organoid media containing growth factors.

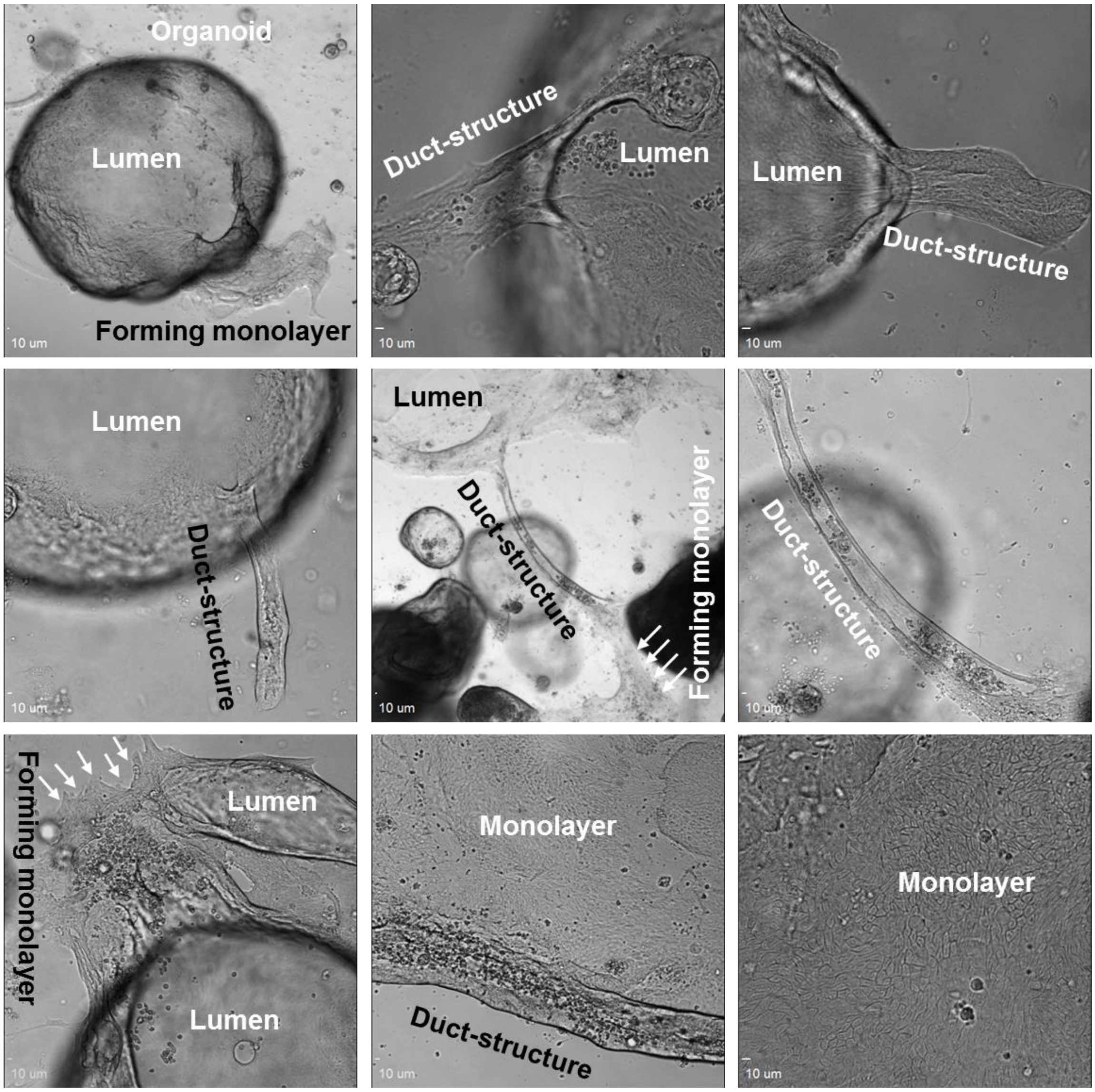

Supplementary Figure 2. Formation of monolayer of PDECs from organoids. Pancreatic ductal organoids grown in Matrigel over time. When the organoids reach the surface, they start forming duct-like structures and PDECs come out from the organoids to form a monolayer.

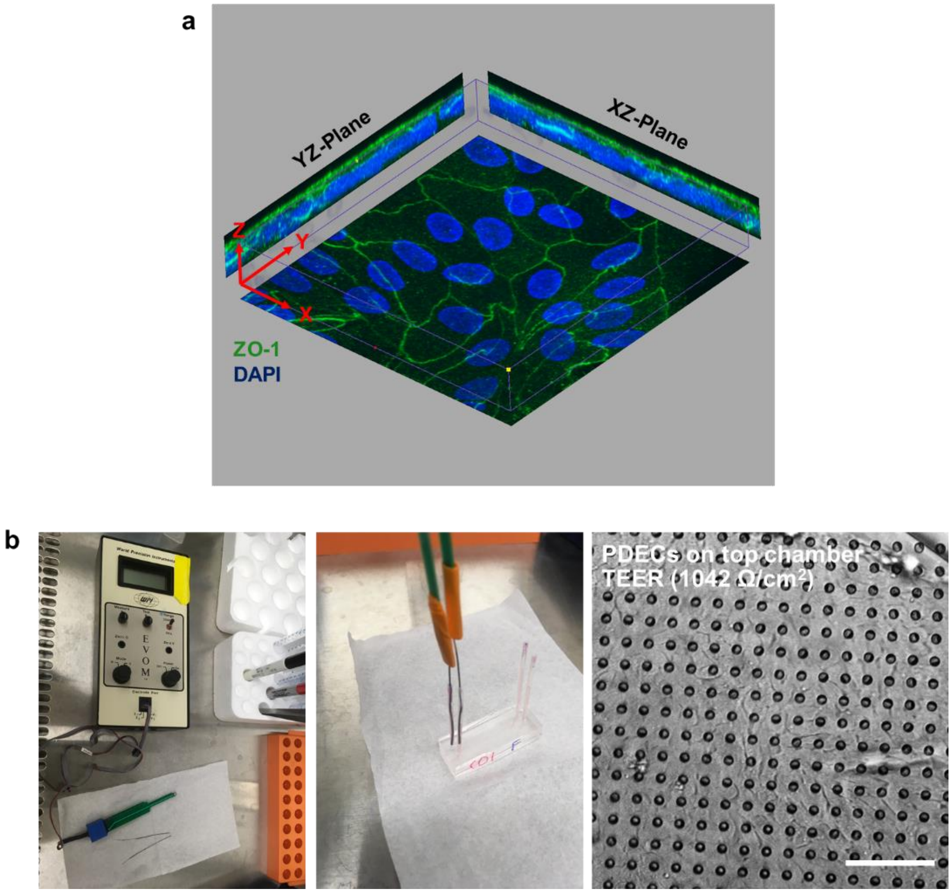

Supplementary Figure 3. Polarized monolayer of PDECs in pancreas-on-a-chip. Polarized monolayer of PDECs on a porous membrane in the chip was verified (**a**) using immunofluorescence image with tight junction, ZO-1, and (**b**) using epithelial volt-ohm meter to measure transepithelial electrical resistance (TEER). The chopstick electrodes were connected to Ag/AgCl wires for the measurement. Scale bar: 100 µm (**b**).

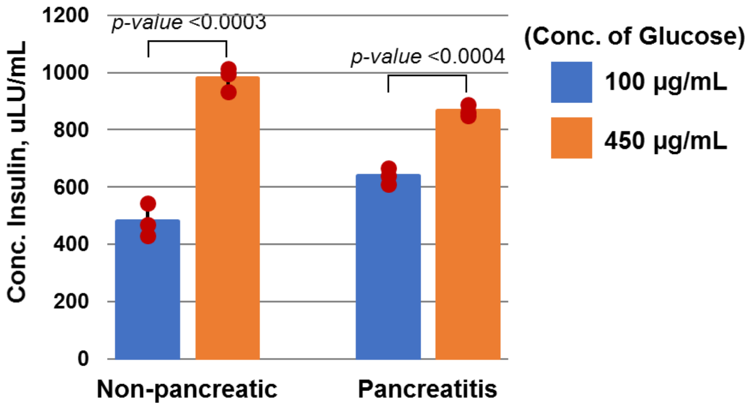

Supplementary Figure 4. Comparison of endocrine function. Comparison of insulin secretion in islet cells from non-pancreatic disease patient and pancreatitis patient. (n = 3 sample preparation from non-pancreatic disease and pancreatitis patient; Data are mean ± SD).

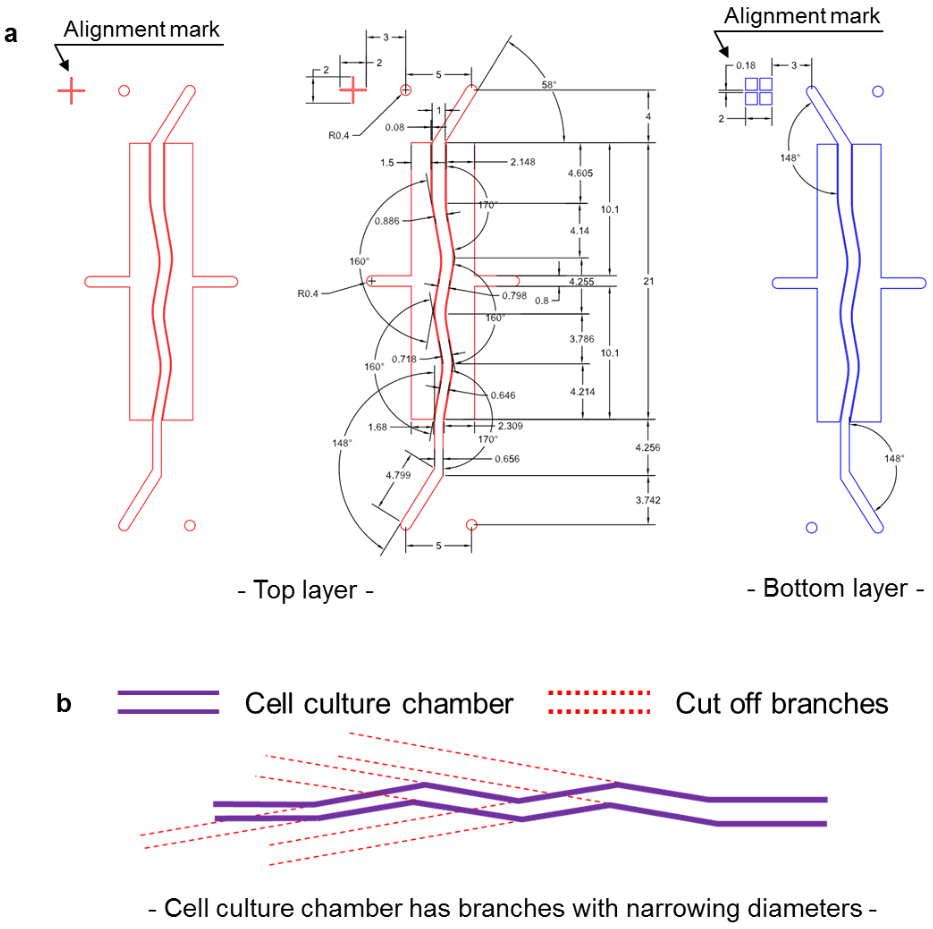

Supplementary Figure 5. Design of cell culture chamber. (**a**) Cell culture chambers in pancreas-on-a-chip was designed using AutoCAD software. (**b**) The chamber has branches with narrowing diameters. (unit: mm).

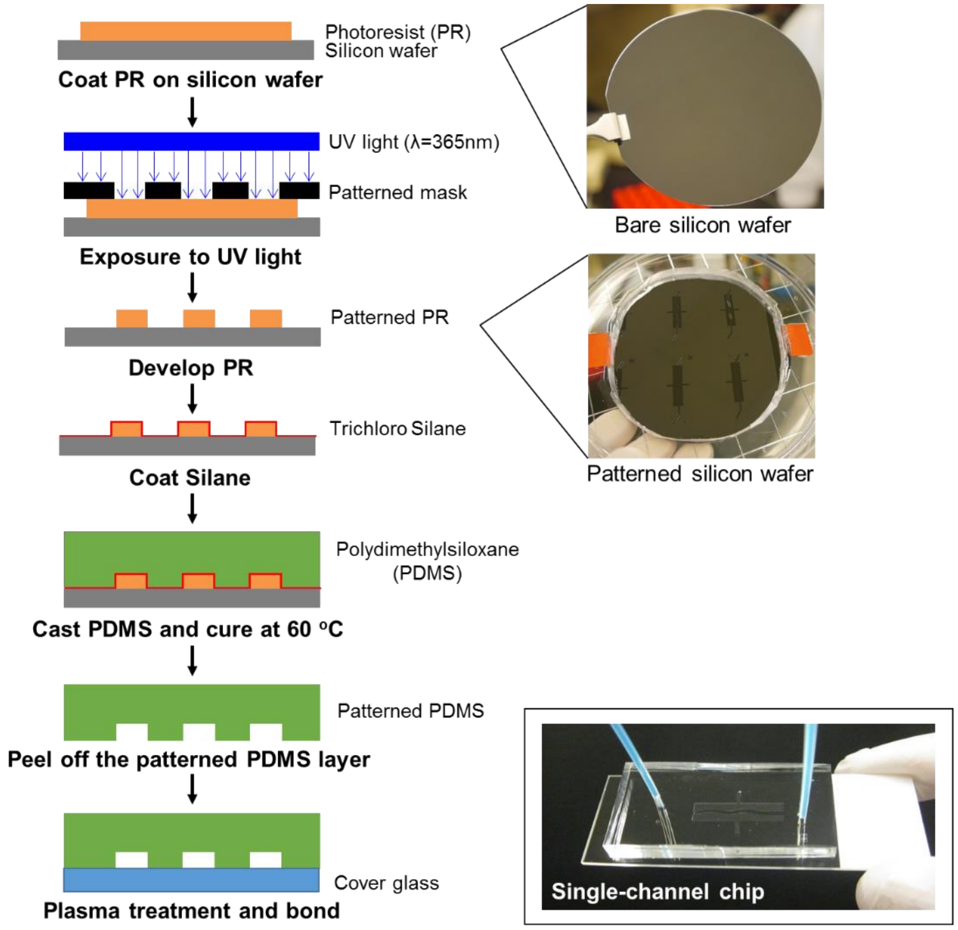

Supplementary Figure 6. Process for single-channel chip. The microfluidic device, single-channel chip, was fabricated using standard photolithography and soft lithography techniques. Initially, designed patterns are created on a silicon wafer through photolithography and cast uncured PDMS to have patterned PDMS layer. Bond the PDMS with glass substrate after treatment with oxygen plasma to seal the chamber.

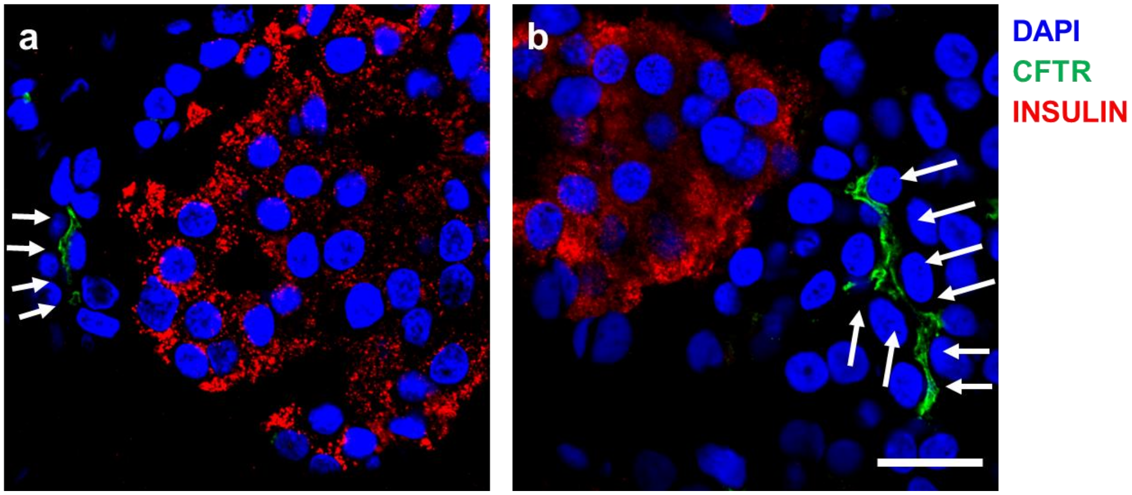

Supplementary Figure 7. Expression of CFTR in pancreatic ductal epithelial cells.
Immunofluorescent microscopy of insulin and CFTR (white arrows) was performed on head of pancreas (**a**) and pancreatic remnant cell pellet followed by TPIAT (**b**). Scale bar: 20 μm.

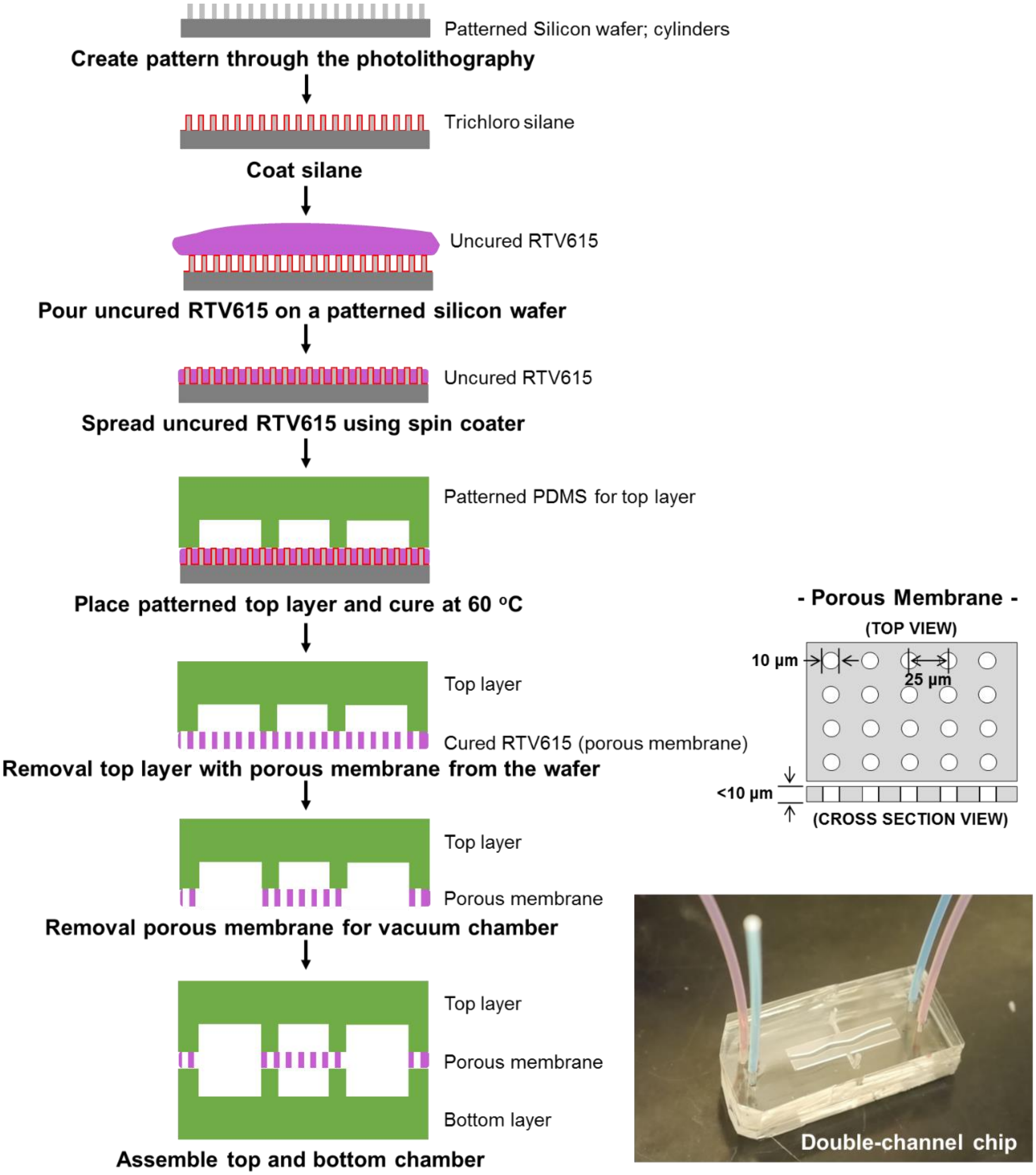

Supplementary Figure 8. Process for double-channel chip. Schematic shows fabrication of pancreas-on-a-chip comprised of two cell culture chambers and a thin layer of porous membrane. Bond patterned PDMS layer for top chamber with porous membrane and alignment with the other PDMS layer for bottom chamber. Pancreas-on-a-chip allows us to co-culture two different types of cells.

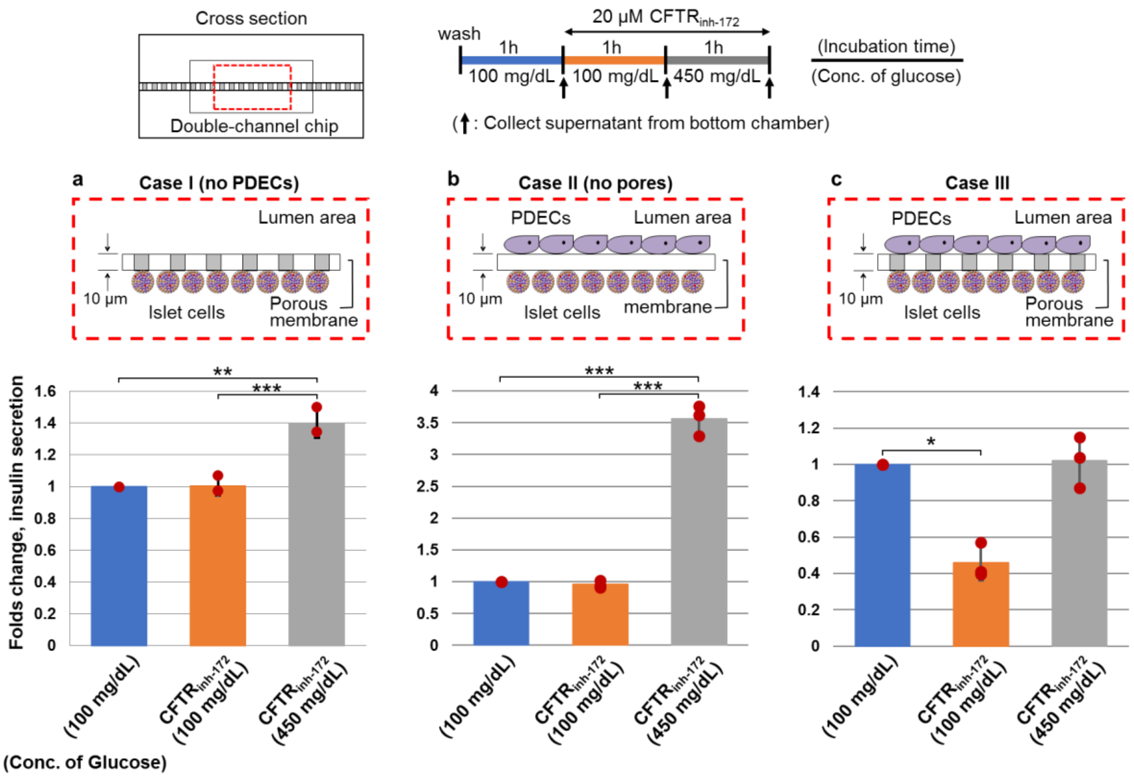

Supplementary Figure 9. Effect of CFTR inhibitor on insulin secretion in double-channel chip. Insulin secretion was monitored from islet cells on the bottom chamber of the double-channel chip followed by incubation with CFTR inhibitor, 20 µM $CFTR_{inh-172}$, applied to the top chamber only. Islet cells were stimulated with high glucose-containing media (450 mg/dL) at the end to verify that endocrine function was not impaired. The CFTR inhibitor did not show any affect on insulin secretion without pancreatic ductal epithelial cells (a; CASE I) or without pores on the membrane (b; CASE II) in the double-channel chip. Insulin secretion was attenuated upon inhibition of CFTR function in double-channel chip with pores (c; CASE III; taken from Figure 5e). (*p-values* from one-way ANOVA and adjust using Bonferroni factor: * < 0.05, ** < 0.005, *** < 0.0005; n = 3 sample preparation from the same patient; Data are mean ± SD).

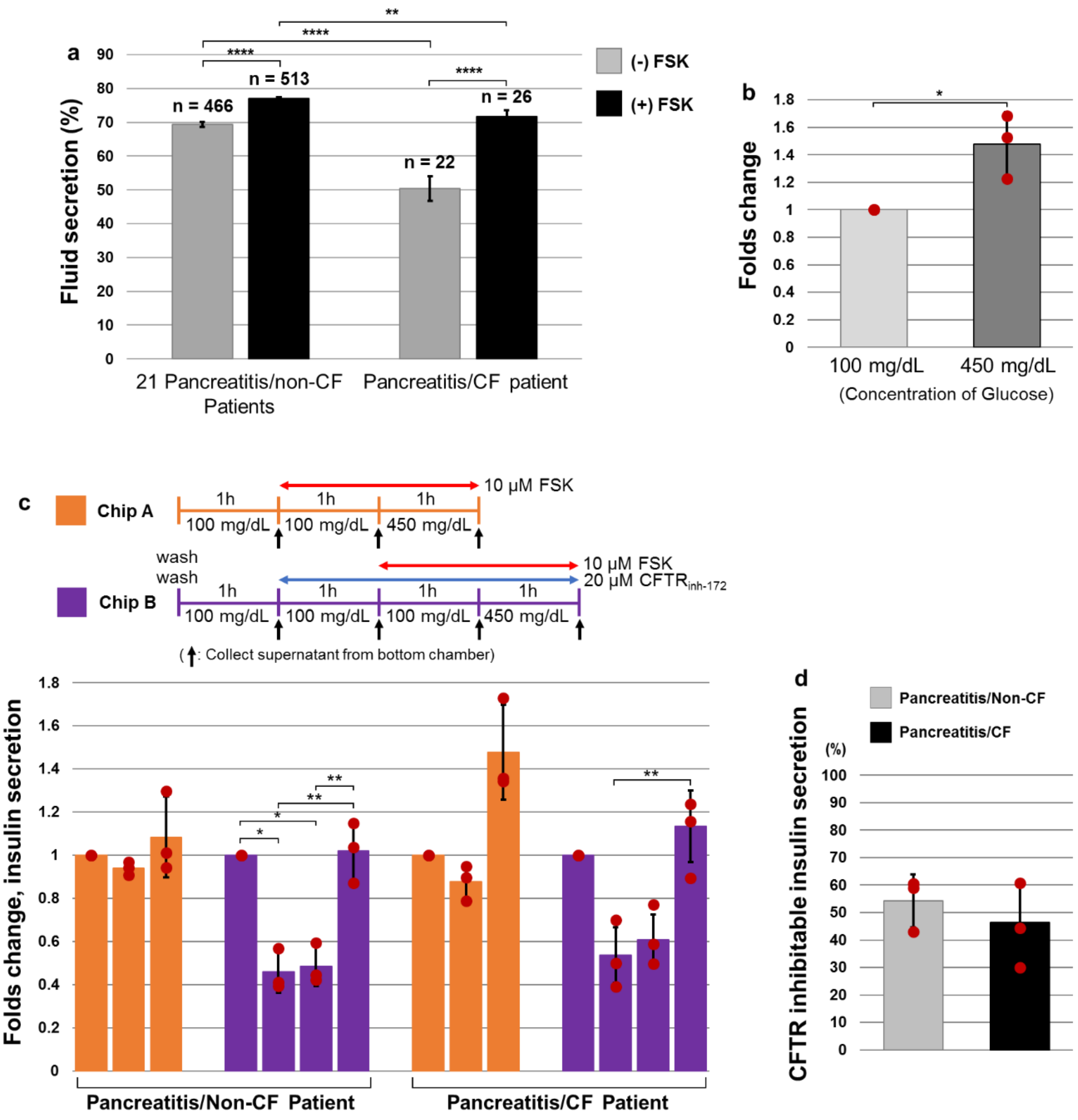

Supplementary Figure 10. Functional examination of PDECs and islet cells obtained from pancreatitis/CF patient. a. CFTR function in PDECs (pancreatitis/CF patient) was observed using fluid secretion measurement and compared with 21 pancreatitis patients. The basal secretion in this patient was 20% lower than pancreatitis patient (n: the number of organoids; Data are mean ± SE). **b.** Endocrine function was monitored using ELISA. Islet cells secreted insulin efficiently in response to highly concentrated glucose (450 mg/dL) (n = 3 sample preparation from the same patient; Data are mean ± SD). **c.** PDECs and islet cells were co-cultured in pancreas-on-a-chip and compared insulin secretion with pancreatitis/non-CF patient **(Fig 5e)** (n = 3; the number of chips; Data are mean ± SD). **d.** Insulin secretion was dramatically decreased by inhibition of CFTR function from both patients, pancreatitis/non-CF patient (54%) and pancreatitis/CF patient (46%) (n = 3; the number of chips; Data are mean ± SD). (*p-values* from one-way ANOVA and adjust using Bonferroni factor: * < 0.05, ** < 0.005, **** < 1.0×10^{-5})

Supplementary Table 1. TPIAT patient summary

Patients	Mutations		Gender	Age (years)	Sweat	FEV1 (%)	BMI
	Allele 1	Allele 2					
Patient 1	SPINK1 (pN34S)	None	F	14	ND	ND	34
Patient 2	CFTR (Δ508)	None	M	8	2	ND	17
Patient 3	None	None	M	13	ND	ND	23
Patient 4	PRSS1 (R122H)	None	F	9	16	ND	18
Patient 5	CFTR (1454G>C Het)	None	F	4	21	ND	15
Patient 6	CFTR (R170H)	SPINK1 (pN34S)	M	18	19	ND	21
Patient 7	None	None	F	13	46	108	25
Patient 8	CPA1	None	F	13	ND	ND	28
Patient 9	CFTR (Δ508), SPINK1	CFTR (R117H)	F	15	51	114	20
