# Supplementary material for: Patient-derived pancreas-on-a-chip to model cystic fibrosis-related disorders
Source: Nat Commun. 2019 Jul 16;10:3124. doi: 10.1038/s41467-019-11178-w (PMC6635497; doi:10.1038/s41467-019-11178-w)
Supplement: Supplementary file 3 — Description of Additional Supplementary Files [file 41467_2019_11178_MOESM3_ESM.pdf]

Description of Additional Supplementary Files

File name: Supplementary Movie 1

Description: Formation of monolayer of PDECs from organoids. Organoids in Matrigel were transferred to a 35-mm dish following breaking down of the Matrigel. The plate was imaged using IncuCyte Zoom system (Essen Bioscience) in a cell culture incubator (5% CO_2, 37°C) and time lapse images were taken every 10 minutes for 26 hours.
