# Supplementary material for: Patient-derived pancreas-on-a-chip to model cystic fibrosis-related disorders
Source: Nat Commun. 2019 Jul 16;10:3124. doi: 10.1038/s41467-019-11178-w (PMC6635497; doi:10.1038/s41467-019-11178-w)
Supplement: Supplementary file 5 — Reporting Summary [file 41467_2019_11178_MOESM5_ESM.pdf]

nature research

Corresponding author(s): Jaimie D. Nathan (jaimie.nathan@cchmc.org) and Anjaparavanda P. Naren (anaren@cchmc.org)

Last updated by author(s): May 31, 2019

Reporting Summary

Nature Research wishes to improve the reproducibility of the work that we publish. This form provides structure for consistency and transparency in reporting. For further information on Nature Research policies, see Authors & Referees and the Editorial Policy Checklist.

Statistics

For all statistical analyses, confirm that the following items are present in the figure legend, table legend, main text, or Methods section.

n/a	Confirmed	
☐	☒	The exact sample size (*n*) for each experimental group/condition, given as a discrete number and unit of measurement
☐	☒	A statement on whether measurements were taken from distinct samples or whether the same sample was measured repeatedly
☐	☒	The statistical test(s) used AND whether they are one- or two-sided<br>*Only common tests should be described solely by name; describe more complex techniques in the Methods section.*
☒	☐	A description of all covariates tested
☐	☒	A description of any assumptions or corrections, such as tests of normality and adjustment for multiple comparisons
☐	☒	A full description of the statistical parameters including central tendency (e.g. means) or other basic estimates (e.g. regression coefficient) AND variation (e.g. standard deviation) or associated estimates of uncertainty (e.g. confidence intervals)
☐	☒	For null hypothesis testing, the test statistic (e.g. *F*, *t*, *r*) with confidence intervals, effect sizes, degrees of freedom and *P* value noted<br>*Give P values as exact values whenever suitable.*
☒	☐	For Bayesian analysis, information on the choice of priors and Markov chain Monte Carlo settings
☒	☐	For hierarchical and complex designs, identification of the appropriate level for tests and full reporting of outcomes
☒	☐	Estimates of effect sizes (e.g. Cohen's *d*, Pearson's *r*), indicating how they were calculated

Our web collection on statistics for biologists contains articles on many of the points above.

Software and code

Policy information about availability of computer code

Data collection	Fluoveiw (Olympus; version 4.1), Slidebook (3I; version 6.0.8), SoftMax Pro (Molecular devices; version 7.0)
Data analysis	Image J (NIH; version 1.51), Microsoft excel

For manuscripts utilizing custom algorithms or software that are central to the research but not yet described in published literature, software must be made available to editors/reviewers. We strongly encourage code deposition in a community repository (e.g. GitHub). See the Nature Research guidelines for submitting code & software for further information.

Data

Policy information about availability of data

All manuscripts must include a data availability statement. This statement should provide the following information, where applicable:

- Accession codes, unique identifiers, or web links for publicly available datasets
- A list of figures that have associated raw data
- A description of any restrictions on data availability

We will provide information (raw unprocessed images and data values for the bar graphs)

Field-specific reporting

Please select the one below that is the best fit for your research. If you are not sure, read the appropriate sections before making your selection.

☒ Life sciences ☐ Behavioural & social sciences ☐ Ecological, evolutionary & environmental sciences

For a reference copy of the document with all sections, see nature.com/documents/nr-reporting-summary-flat.pdf

Life sciences study design

All studies must disclose on these points even when the disclosure is negative.

Sample size	All the quantitative experiments were performed at least n=3 sample size and student t-test was used for pair-wise comparison.
Data exclusions	No data point was excluded from the quantitative analysis.
Replication	All the experiments were performed in triplicates independently. Some of the experiments were performed by different experimenters in a blinded fashion to ensure reproducibility of the data.
Randomization	Same cells were used to perform different treatments. The samples were collected in random order only known to the experimenter and were read by different lab personnel.
Blinding	Some of the experiments were performed by different experimenters in a blinded fashion to ensure reproducibility of the data.

Reporting for specific materials, systems and methods

We require information from authors about some types of materials, experimental systems and methods used in many studies. Here, indicate whether each material, system or method listed is relevant to your study. If you are not sure if a list item applies to your research, read the appropriate section before selecting a response.

Materials & experimental systems

n/a	Involved in the study	
☐	☒	Antibodies
☒	☐	Eukaryotic cell lines
☒	☐	Palaeontology
☒	☐	Animals and other organisms
☒	☐	Human research participants
☒	☐	Clinical data

Methods

n/a	Involved in the study	
☒	☐	ChIP-seq
☒	☐	Flow cytometry
☒	☐	MRI-based neuroimaging

Antibodies

Antibodies used	Primary antibodies: anti-CFTR R1104 (Eric Sorscher lab, CF Center, University of Alabama, Birmingham, Alabama, USA [presently, Emory University, Atlanta, Georgia, USA]), anti-ZO-1 (BD Biosciences; #610967), anti-ENaC (Invitrogen; #PA1-920A), anti-Cytokeratin 19 (Invitrogen; #MA5-12663), anti-E cadherin (Cell Signaling Technology; #3195), anti-insulin (Cell Signaling; #C27C9), and anti-glucagon (Sigma; #G2654). Secondary antibodies: Alexa Fluor 488 or 568 (Invitrogen). Alexa Fluor 488 Phalloidin (Invitrogen; #A12379) and DAPI (Invitrogen; #D1306) were used for immunofluorescence microscopy.
Validation	The antibodies were validated using western blotting and in some cases using purified proteins. Also negative control were run each time for the experiments.
